# The EffecTs of Amlodipine and other Blood PREssure Lowering Agents on Microvascular FuncTion in Small Vessel Diseases (TREAT-SVDs) trial: Study protocol for a randomised crossover trial

**DOI:** 10.1177/23969873221143570

**Published:** 2022-12-16

**Authors:** Anna Kopczak, Michael S Stringer, Hilde van den Brink, Danielle Kerkhofs, Gordon W Blair, Maud van Dinther, Laurien Onkenhout, Karolina A Wartolowska, Michael J Thrippleton, Marco Duering, Julie Staals, Martin Middeke, Elisabeth André, Bo Norrving, Marie-Germaine Bousser, Ulrich Mansmann, Peter M Rothwell, Fergus N Doubal, Robert van Oostenbrugge, Geert Jan Biessels, Alastair JS Webb, Joanna M Wardlaw, Martin Dichgans

**Affiliations:** 1 Institute for Stroke and Dementia Research, University Hospital, LMU Munich, Munich, Germany; 2 Centre for Clinical Brain Sciences, University of Edinburgh, Edinburgh, UK; 3 Department of Neurology, UMC Utrecht Brain Center, University Medical Center Utrecht, Utrecht, The Netherlands; 4 Department of Neurology and School for cardiovascular diseases (CARIM), Maastricht University Medical Center+, Maastricht, The Netherlands; 5 Wolfson Centre for Prevention of Stroke and Dementia, Nuffield Department of Clinical Neurosciences, University of Oxford, Oxford, UK; 6 Medical Image Analysis Center (MIAC AG) and Department of Biomedical Engineering, University of Basel, Basel, Switzerland; 7 Hypertoniezentrum München, Excellence Centre of the European Society of Hypertension (ESH), Munich, Germany; 8 Münchner Studienzentrum, Faculty of Medicine, Technical University Munich (TUM), Munich, Germany; 9 Neurology, Department of Clinical Sciences Lund, Lund University, and Neurology, Skåne University Hospital Lund/Malmö, Sweden; 10 Hôpital Lariboisière, APHP, Université Paris-Cité, Paris, France; 11 Institute for Medical Information Processing, Biometry, and Epidemiology, LMU Munich, Munich, Germany; 12 UK Dementia Research Institute, University of Edinburgh, Edinburgh, UK; 13 Munich Cluster for Systems Neurology (SyNergy), Munich, Germany; 14 German Center for Neurodegenerative Diseases (DZNE), Munich, Germany

**Keywords:** Small vessel diseases, lacunar stroke, vascular cognitive impairment, CADASIL, cerebrovascular reactivity, blood pressure variability, antihypertensive drug classes, amlodipine, magnetic resonance imaging, randomised clinical trial

## Abstract

**Background:**

Hypertension is the leading modifiable risk factor for cerebral small vessel diseases (SVDs). Yet, it is unknown whether antihypertensive drug classes differentially affect microvascular function in SVDs.

**Aims:**

To test whether amlodipine has a beneficial effect on microvascular function when compared to either losartan or atenolol, and whether losartan has a beneficial effect when compared to atenolol in patients with symptomatic SVDs.

**Design:**

TREAT-SVDs is an investigator-led, prospective, open-label, randomised crossover trial with blinded endpoint assessment (PROBE design) conducted at five study sites across Europe. Patients aged 18 years or older with symptomatic SVD who have an indication for antihypertensive treatment and are suffering from either sporadic SVD and a history of lacunar stroke or vascular cognitive impairment (group A) or CADASIL (group B) are randomly allocated 1:1:1 to one of three sequences of antihypertensive treatment. Patients stop their regular antihypertensive medication for a 2-week run-in period followed by 4-week periods of monotherapy with amlodipine, losartan and atenolol in random order as open-label medication in standard dose.

**Outcomes:**

The primary outcome measure is cerebrovascular reactivity (CVR) as determined by blood oxygen level dependent brain MRI signal response to hypercapnic challenge with change in CVR in normal appearing white matter as primary endpoint. Secondary outcome measures are mean systolic blood pressure (BP) and BP variability (BPv).

**Discussion:**

TREAT-SVDs will provide insights into the effects of different antihypertensive drugs on CVR, BP, and BPv in patients with symptomatic sporadic and hereditary SVDs.

**Funding:**

European Union’s Horizon 2020 programme.

**Trial registration:**

NCT03082014.

## Introduction

Stroke and dementia rank among the most pressing health issues in Europe.^1,2^ Cerebral small vessel diseases (SVDs) have emerged as a central link between these two major co-morbidities.^3,4^ SVDs account for up to 30% of strokes and contribute to at least 40% of dementia cases.^3^ Yet, there is no specific treatment with proven efficacy against SVDs.

SVDs can be separated into sporadic and hereditary forms.^4^ Hypertension remains the leading modifiable risk factor for SVDs including Cerebral Autosomal Dominant Arteriopathy with Subcortical Infarcts and Leukoencephalopathy (CADASIL), the most common hereditary type of SVD.^4,5^ Current treatment guidelines recommend blood pressure (BP) control for covert SVD and secondary stroke prevention including for patients with lacunar stroke.^6,7^ To date, there have been few randomised controlled trials (RCTs) on blood pressure lowering in patients with lacunar stroke or other clinical manifestations of SVD.^8,9^

Data from RCTs in hypertensive patients point towards a differential effect of antihypertensive drug classes on the risk of stroke.^10–13^ This may relate to the differential effect of antihypertensive drug classes on blood pressure variability (BPv), which has been identified as an independent risk factor for stroke^14,15^ and dementia.^16^ BPv was further shown to be associated with the presence or progression of white matter hyperintensities (WMH) as a marker of SVD on brain magnetic resonance imaging (MRI).^17^ In a meta-analysis of RCTs of BP lowering drugs, BPv was shown to be reduced by calcium channel blockers (CCBs) and increased by angiotensin-converting enzyme inhibitors, angiotensin-receptor blockers, and beta-blockers in ascending order.^18^ Also, a recent Mendelian randomisation study found that genetic proxies for CCBs showed particularly strong associations with small vessel stroke and the related radiologic phenotype of WMH.^19^ However, there have been no RCTs that have compared different classes of antihypertensive drugs in patients with lacunar stroke or other manifestations of SVD.

Recent experimental data suggest a differential effect of antihypertensive drug classes on microvascular function in the brain. The CCB amlodipine was found to have a favourable effect on functional hyperaemia in chronically hypertensive mice when compared to losartan^20^ but whether antihypertensive drug classes differentially affect microvascular function in human SVDs remains unknown. Microvascular function can be measured non-invasively in humans by assessing cerebrovascular reactivity (CVR).^21^ CVR can be quantified by blood oxygen level dependent (BOLD) MRI during inhalation of carbon dioxide (CO_2_) versus air^22^ and has been shown to be impaired after lacunar stroke.^23^ To assess the effect of CCBs and other antihypertensive drug classes on microvascular function in SVDs, we initiated the ‘Effec**T**s of Amlodipine and other Blood P**RE**ssure Lowering **A**gents on Microvascular Func**T**ion in **S**mall **V**essel **D**isease**s**’ (TREAT-SVDs) trial.

## Methods

### Study design

TREAT-SVDs (ClinicalTrials.gov identifier: NCT03082014; EudraCT number 2016-002920-10) is a multinational phase IIIb clinical trial. The study is conducted as a prospective, randomised, open-label, crossover study with blinded endpoint assessment (PROBE design).^24^ The pre-defined primary study objective is to test the hypothesis that amlodipine has a beneficial effect on microvascular function in patients with SVDs when compared to either losartan or atenolol. The pre-defined secondary study objective is to test the hypothesis that losartan has a beneficial effect on microvascular function in patients with SVDs when compared to atenolol.

### Study setting

TREAT-SVDs collaborators are based at five study sites in Europe (Supplemental Figure 1): Ludwig-Maximilians-Universität Munich, Germany; University of Oxford, UK; University of Edinburgh, UK; University of Utrecht, The Netherlands; and University of Maastricht, The Netherlands. All study-related procedures are conducted at these five sites. Participant information centres at the University of Glasgow, UK, and the University of Leiden, The Netherlands, inform eligible patients with CADASIL about the study but do not contribute to the study visits. The University Hospital Munich (‘Klinikum der Universität München’) serves as sponsor of the TREAT-SVDs trial. The study was approved by the respective local ethics committees and regulatory authorities. All participants provide written informed consent.

### Eligibility criteria

TREAT-SVDs includes patients aged 18 years or older with symptomatic SVD who have an indication for antihypertensive treatment and are suffering from either sporadic SVD (group A) or CADASIL (group B).

**Figure 1.**
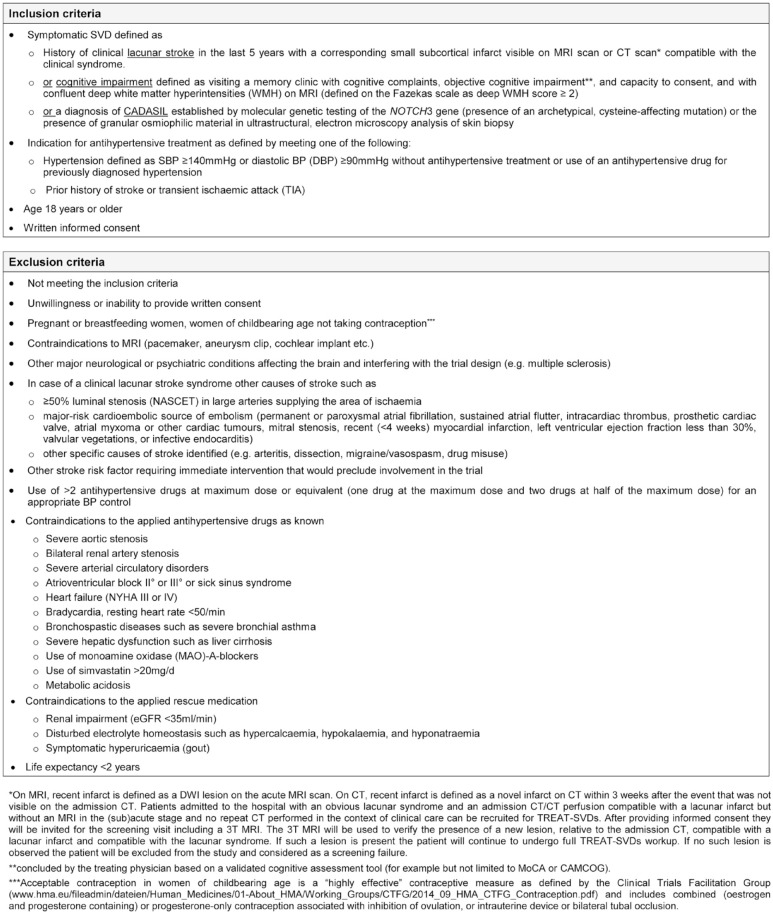
Inclusion and exclusion criteria for the TREAT-SVDs trial.

Patients with sporadic SVD are eligible for study inclusion if they have a history of lacunar stroke or vascular cognitive impairment. Sporadic patients with lacunar stroke are required to have a subcortical infarct compatible with the clinical syndrome and visible as an acute diffusion weighted imaging-positive lesion on MRI or as a novel subcortical infarct on computed tomography (CT) scan repeated within 3 weeks after stroke onset if not visible on the admission CT. The maximum time interval between stroke and study inclusion is 5 years. Patients with other causes of stroke are excluded (Figure 1). Sporadic patients with vascular cognitive impairment are eligible for study inclusion if they are visiting a memory clinic with cognitive complaints and are diagnosed with objective cognitive impairment as documented by a validated cognitive assessment tool. They must further have deep WMH defined on the Fazekas scale as deep WMH score ⩾2.

Patients with CADASIL must have a definite diagnosis of CADASIL as documented by genetic testing of the *NOTCH3* gene (presence of an archetypical, cysteine-affecting mutation) or ultrastructural examination of a skin biopsy demonstrating granular osmiophilic material within microvessels.

Study participants must have an indication for antihypertensive treatment meeting one of the following criteria: (i) hypertension defined as systolic BP (SBP) ⩾140 mmHg or diastolic BP (DBP) ⩾90 mmHg or use of an antihypertensive drug for the treatment of hypertension; or (ii) a history of stroke or TIA.

Patients with severe hypertension defined as taking more than the equivalent of two antihypertensive drugs at maximum dose (e.g. one drug at the maximum dose and two drugs at half of the maximum dose) for appropriate BP control are excluded. Patients who lack capacity to consent cannot be included. Detailed inclusion and exclusion criteria are shown in Figure 1.

### Interventions

Antihypertensive treatment is given as open-label oral medication in standard dose. Trial medications are amlodipine (standard dose: 5 mg), losartan (standard dose: 50 mg), and atenolol (standard dose: 50 mg). The trial drugs applied in this clinical trial are ‘Medicinal products’ according to Article 1(2) of Directive 2001/83/EC. All applied trial drugs are approved for the treatment of hypertension, recommended in national and international guidelines and not under patent protection. Local pharmacies buy commercially available products and medication is provided to study participants with an open label. Dispensation of trial medication is documented in a drug accountability form.

Eligible patients are randomly assigned to one of the three treatment arms, starting with either amlodipine, losartan or atenolol by a computer-generated multi-block randomisation with 1:1:1 allocation stratified for the study site and for sporadic SVD patients (group A) and CADASIL patients (group B). Randomisation is performed centrally at the Münchner Studienzentrum (MSZ) by an independent biometrician for all study participants prior to study inclusion.

**Figure 2.**
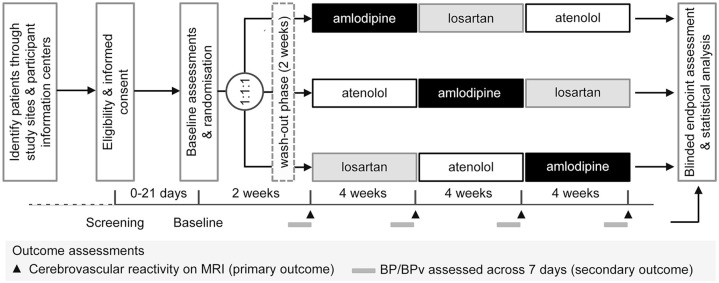
Study design of the TREAT-SVDs trial. TREAT-SVDs uses a PROBE (prospective, randomised, open-label, blinded endpoint assessment) design. Participants are randomised with a 1:1:1 treatment allocation. The primary outcome measure is cerebrovascular reactivity (CVR) as determined by the change in BOLD MRI brain scan signal in response to a hypercapnic challenge after the 2 weeks wash-out phase and after 4 weeks of monotherapy while still on medication. Secondary outcome measures include the mean systolic blood pressure (BP) and blood pressure variability (BPv) as assessed by daily telemetric monitoring within the last week of the run-in phase and within the last week of each treatment phase. For further details see text.

The three sequences of drug intake are shown in Figure 2. At the beginning of the trial, patients stop taking their regular antihypertensive medication for a 2-week run-in phase. During this phase, participants are prohibited to take antihypertensive drugs except for thiazide or thiazide-like diuretics such as hydrochlorothiazide or bendroflumethiazide, which serve as rescue medication. BP is measured during the whole trial.

The trial drugs are taken in the morning upon rising, each one for 4 weeks of monotherapy according to the randomised sequence of drug intake. The respective study physician is responsible for adjusting the dosage of the trial drug; rescue medication is taken as needed. The treatment aim is to lower SBP to <140 mmHg and DBP to <90 mmHg. Switching between BP lowering agents is done directly without washout. Participants return unused tablets of the trial medication at each follow-up visit. Unused tablets are counted and documented in the drug accountability form. Antihypertensive medication other than the trial drugs are not allowed. Concomitant medication is assessed and documented during the trial as described in the Supplemental Methods. The regular duration of intervention per patient is 14 weeks.

### Primary outcome

**Figure 3.**
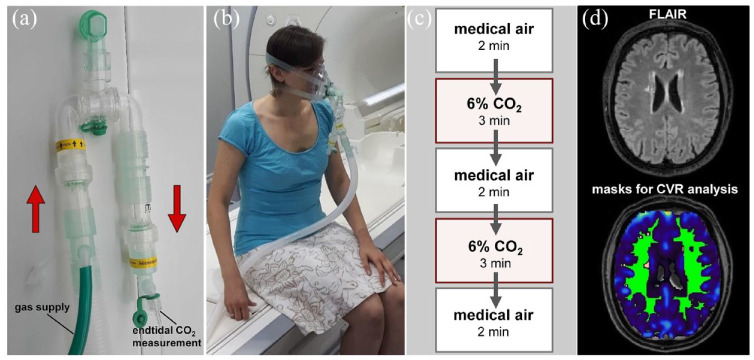
Assessment of the primary outcome measure in TREAT-SVDs trial. Shown are the technical set-up of the breathing circuit for application of the hypercapnic challenge and measurement of end tidal CO_2_ (a), the fixed breathing circuit before positioning in the MRI scanner enabling continuous and controlled breathing of medical air or 6% CO_2_ for quantification of cerebrovascular reactivity (CVR) (b), the breathing paradigm with alternating medical air and 6% CO_2_ (c), and an exemplary FLAIR scan slice from a study participant with the corresponding masks for CVR analysis (d). The primary endpoint is change in CVR from baseline to treatment in normal-appearing white matter (green area). Further analyses include CVR in white matter hyperintensities (yellow area) and in subcortical grey matter.

The primary outcome measure is CVR as determined by BOLD brain MRI signal response to hypercapnic challenge at the end of the 2-week run-in phase and at the end of each 4 weeks period of drug treatment. CVR is measured at 3T using two blocks of breathing 6% CO_2_ in medical air for 3 min, alternating with medical air, delivered through a close-fitting anaesthetic facemask (Figure 3).^25,26^ End tidal CO_2_ is recorded throughout the 12-min paradigm. The methodology was piloted in the multicentre study INVESTIGATE-SVDs.^26^

BOLD MRI data is processed and matched with the end tidal CO_2_ records centrally at the University of Edinburgh according to a standard operating procedure.^25,26^ CVR is calculated by fitting the percentage BOLD signal change to a linear model with end tidal CO_2_ as a predictor.^25^ Patients also have structural sequences (T1w, FLAIR, T2w, dMRI, SWI/FLASH) to assess brain volume, WMH volume, and other SVD features. All MRI scans are performed using a harmonised acquisition protocol.^26^ Acquisition parameters are listed in Supplemental Tables 1–2.

CVR is assessed in multiple pre-specified brain regions, for example, in normal appearing white matter (NAWM), WMH and in subcortical grey matter. Change in CVR from baseline to treatment is calculated for each study drug to compare drug effects. The primary endpoint is change in CVR in NAWM from baseline to treatment. Further analyses include change in CVR in WMH from baseline to treatment, and change in CVR in subcortical grey matter from baseline to treatment. Analyses are performed by experienced raters blinded to the clinical status and information related to trial medication.

### Secondary outcomes

Secondary outcome variables include i) mean SBP assessed by daily telemetric monitoring and ii) BPv operationalised as coefficient of variation (100 × standard deviation/mean SBP) assessed by daily telemetric monitoring within the last week of the run-in phase and the last week of each treatment phase.

**Figure 4.**
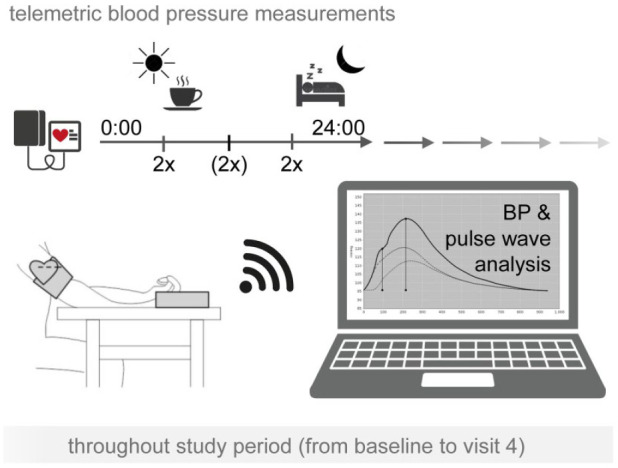
Blood pressure measurement and pulse wave analysis in the TREAT-SVDs trial. Shown are the time points of blood pressure (BP) measurement as secondary outcome measure in the TREAT-SVDs trial.

Participants are asked to measure their BP at least twice daily (Figure 4). The morning measures after awakening and the evening measures before going to bed are mandatory^27^ while the BP measurement around noon is optional. Study participants are instructed to repeat BP measurements within 5 min so that two consecutive readings are available at each time point. For data analyses, only the second BP measurement from the paired reading will be used unless there is only a single BP recording, in which case this one will be used for analysis. The BP device further allows for pulse wave analysis to derive pulse wave velocity and central BP.

### Clinical assessments

At baseline, clinical status is assessed by the National Institutes of Stroke Scale (NIHSS) and the modified Rankin Scale (mRS). Cognitive status is measured by the ‘consortium to establish a registry for Alzheimer’s disease’ (CERAD) neuropsychological test battery to which phonematic fluency, trail making test A and B, and the digit span forward and backward are added to allow for better detection of vascular cognitive impairment. Detailed information on cognitive tests is described elsewhere.^26,28^ Laboratory investigations are listed in the Supplemental Methods.

### Sample size

A total of 49 sporadic SVD patients are needed to detect an effect that leads to a 0.1% absolute difference in CVR (standard deviation 0.21) with a power of 90% on a 5% significance level using a two-sided one sample *t*-test. This implies that 17 patients are allocated in each of the three sequences. Assuming a drop-out of eight patients per sequence, a total of 75 sporadic patients will be included in the cross-over trial.

A total of 28 patients are needed to detect an effect that leads to a 0.1% absolute increase in CVR (standard deviation 0.18) with a power of 80% on a 5% significance level using a two-sided one sample *t*-test. Therefore, a total of 30 CADASIL patients will be included. In total, we aim to include 105 study participants (75 patients with sporadic SVDs, 30 CADASIL patients) into the trial.

### Recruitment

Potentially eligible patients are identified in the everyday clinical practice of research staff or referred to them for assessment of eligibility having been identified elsewhere (e.g. by participant information centres). Collaborators screen new admissions to hospital or outpatient clinics and may identify patients looked after by their service for lacunar stroke, vascular cognitive impairment or CADASIL. The minimum time interval between lacunar stroke and study inclusion is 1 month. The study investigator is responsible for confirming eligibility, ensuring informed consent is obtained and that the informed consent form is completed, signed and dated by all parties.

### Allocation

**Figure 5.**
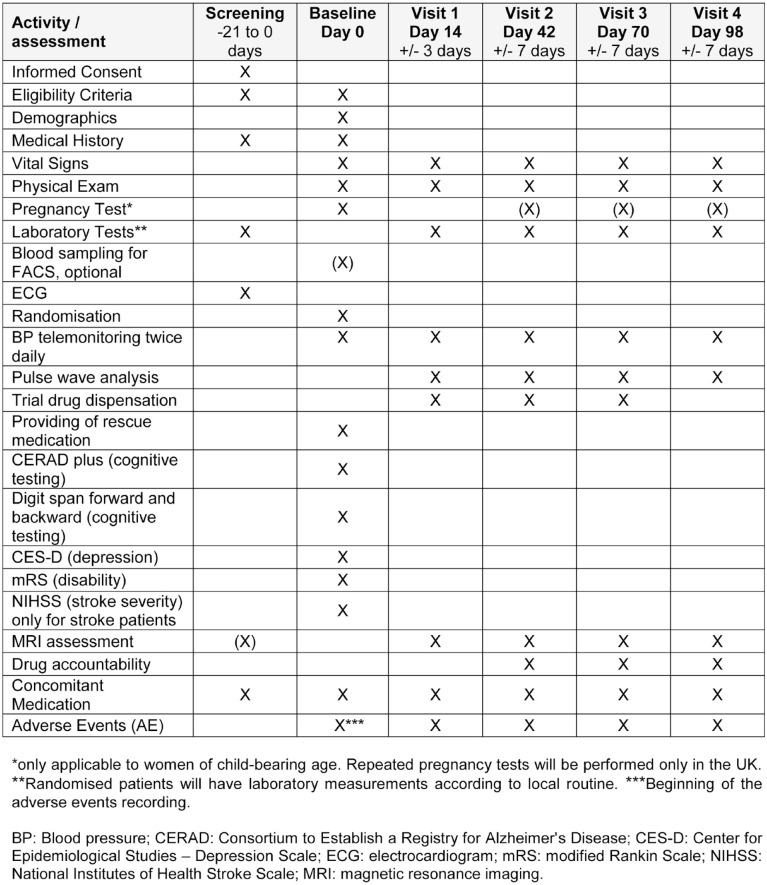
Schedule of enrolment, interventions, and assessments in the TREAT-SVDs trial.

Having obtained consent, the screening visit takes place. This visit can be done at the day of the baseline assessments (Figure 5) but not earlier than 3 weeks prior to baseline. If eligible, patients are randomised by opening the envelope with the respective patient ID that is stored in the investigator site file at each study site at the end of the baseline visit.

### Data management

Data is collected on worksheets and entered in an electronic case report form. Data management is done by the MSZ, which uses the commercial trial software (MACRO^®^). The trial database was developed and validated before data entry based on standard operating procedures at the MSZ. Data are entered online at the trial sites. Plausibility checks are run during data entry, thereby detecting potential discrepancies in real-time. The MSZ’s data management team conducts further checks for completeness and plausibility and clarifies any questions with the trial sites electronically via the trial software as part of the regular remote monitoring. Electronic queries have to be answered by the trial site without unreasonable delay. Further details are specified in the data management plan. All changes made to the data are documented in an audit trail.

### Statistical analysis

A linear mixed effects (LME) model will be employed to assess sequence effects in the crossover and corresponding treatment effects. The primary analysis follows a hierarchical test principle (closed testing procedure) by assessing a global effect between atenolol, amlodipine, and losartan. In case of a significant difference, the analysis will assess the three pairs of differences. All analyses will be conducted on three trial populations: (1) intention-to-treat population (all trial subjects enrolled into the trial and randomised), (2) per protocol population (all trial subjects who were treated according to protocol and reached all primary endpoints) and (3) the safety population (all trial subjects who received any study drug or other trial treatment).

The analysis will be stratified by patients with sporadic SVDs (group A) and patients with CADASIL (group B). Group A will be the primary group for analysis; group B will allow to check for a common treatment effects. Additional sensitivity analyses will be performed as appropriate. The detailed analyses is described in a statistical analysis plan (SAP). All analyses will be conducted using R (R Project for Statistical Computing, Vienna, Austria). A *p-*value of <0.05 will be considered statistically significant.

### Safety Monitoring Board (SMB)

Safety and the risk-benefit-ratio of study participation is assessed regularly every 3 months by the sponsor and the SMB, which is independent of the sponsor, consists of experts in clinical trials (MGB, BN) and meets together with the ethical advisor on an annual basis. During the period of recruitment into the study, data on the number of study participants including reasons for withdrawal, if applicable, as well as on type of adverse events, adverse reactions, serious adverse events, serious adverse reactions, and serious unexpected adverse reactions are supplied in strict confidence to the members of the SMB and the sponsor. In light of these analyses, the SMB advise the sponsor if, in their view, there is still a reasonable risk-benefit ratio and if action including termination of the trial is needed.

### Harms

TREAT-SVDs is a RCT involving antihypertensive drugs with well-established safety profiles. After enrolment, patients with SVDs that are in need of an antihypertensive treatment have to stop taking their antihypertensive drugs for the 2-week run-in period. This duration was chosen because it ensures a complete wash-out of the most frequently used antihypertensive drugs while keeping the period without antihypertensive drugs as short as possible.

During this run-in period, there is an increased risk of hypertensive crisis and cardiovascular events. To prevent this, patients taking more than two antihypertensive drugs at maximum dose are excluded. Second, participants are measuring their BP regularly during the whole trial period, and BP is monitored by the investigators remotely with alarms for extreme values. Third, rescue medication is administered as needed. Finally, study participants exhibiting persisting hypertension despite antihypertensive treatment according to the trial protocol are withdrawn from the study.

Risks within the study have been stratified according the Risk Assessment and Categorisation Tool (RACT). The overall risk was assessed to be low. The sponsor (‘Klinikum der Universität München’) has insurance in place (which includes default compensation) for negligent harm caused by poor protocol design by employees of their university hospital. Sites participating in the study will be liable for clinical negligence and other negligent harm to individuals taking part in the study and covered by the duty of care owed to them by the sites concerned.

### Monitoring and auditing

The TREAT-SVDs internal monitoring procedure to assure appropriate conduct of the trial uses a combination of onsite and remote monitoring unless issues are identified that can only be addressed by site monitoring in accordance with the Monitoring Plan agreed by the sponsor. This is regularly reviewed during the course of the trial. The Audit Plan is available from the corresponding author upon reasonable request.

### Steering committee

All lead investigators are steering committee members. The steering committee agrees with the final protocol, reviews the progress of the study, agrees to changes to the protocol, and decides upon further research proposals on TREAT-SVDs.

### Legislation and guidelines

At each trial site, the clinical trial started after approval of the competent local ethics committee concerning the suitability of the trial site and the qualifications of the investigators. The trial was submitted to and approved by the appropriate independent research ethics committee for each participating centre, prior to entering any subject into the trial. Detailed information is presented in the Supplemental Methods. The trial is conducted according to EU Directive 2001/20/EC and the implementation into national laws. The present trial protocol and any amendments were and will be prepared in accordance with the Declaration of Helsinki as amended in Fortaleza (2013).

### Role of the funding source

This study is funded by the European Union’s Horizon 2020 programme. The funding source had no role in the design of this study and will not have any role during its execution, analyses, interpretation of the data, or decision to submit results.

## Discussion

TREAT-SVDs is a prospective, multicentre trial that aims to assess the effects of amlodipine, losartan, and atenolol on microvascular function in patients with sporadic and hereditary forms of SVDs. The primary study hypothesis is that amlodipine has a beneficial effect on microvascular function in patients with SVDs when compared to either losartan or atenolol. The secondary study hypothesis is that losartan has a beneficial effect when compared to atenolol. To our knowledge, there is no other RCT underway that would address these questions. Enrolment in TREAT-SVDs commenced in February 2018. Recruitment is ongoing with 101 participants enrolled by 01/10/2022 and is projected to end in December 2022.

Our primary outcome measure is CVR which is assessed centrally by experienced raters blinded to all other data. Local study personnel were trained centrally on how to perform CVR measurements enabling harmonisation of study procedures and allowing for a high comparability of measures across study sites. Our secondary outcome measure is mean systolic BP and BPv. To assess the secondary outcome, all study participants are using a certified telemetric BP device that measures BP and performs pulse wave analysis. BP data is transferred to the study site in real-time, and BP is monitored remotely during the whole study period.

The TREAT-SVDs trial is designed as a randomised three-sequence crossover study. With this study design, it is possible to compare drug effects of amlodipine, losartan, and atenolol in each individual participant thereby reducing confounders. However, the trial is demanding due to the subsequent intake of three different trial drugs and four MRI scans including hypercapnia. The period of a 4-week trial drug intake was chosen to detect a stable pharmacological effect while also maintaining participants’ adherence to the trial medication. However, we will not be able to investigate long-term effects of the applied antihypertensive drugs. Other potential limitations are that patients with severe hypertension taking more than two antihypertensive drugs at a maximum dose or equivalent are excluded from study participation for safety reasons with respect to the 2-week wash-out period. Also, recruitment of CADASIL patients is challenging since CADASIL is a rare disease, and CADASIL patients are younger and less often have hypertension. Nevertheless, investigating drug effects in CADASIL is important since arterial hypertension is also the leading modifiable risk factor in CADASIL patients. As an important aspect, the inclusion of CADASIL patients will allow to compare sporadic and hereditary forms of SVDs.

In conclusion, TREAT-SVDs will provide first insights from a clinical trial into the effects of different antihypertensive drug classes on microvascular function in sporadic and hereditary forms of SVDs. In addition, the trial provides proof of concept for the feasibility of multicentre-trials involving serial MRIs with hypercapnic challenge to investigate drug effects in patients with symptomatic SVDs.

## Supplemental Material

sj-docx-1-eso-10.1177_23969873221143570 – Supplemental material for The EffecTs of Amlodipine and other Blood PREssure Lowering Agents on Microvascular FuncTion in Small Vessel Diseases (TREAT-SVDs) trial: Study protocol for a randomised crossover trial Click here for additional data file. Supplemental material, sj-docx-1-eso-10.1177_23969873221143570 for The EffecTs of Amlodipine and other Blood PREssure Lowering Agents on Microvascular FuncTion in Small Vessel Diseases (TREAT-SVDs) trial: Study protocol for a randomised crossover trial by Anna Kopczak, Michael S Stringer, Hilde van den Brink, Danielle Kerkhofs, Gordon W Blair, Maud van Dinther, Laurien Onkenhout, Karolina A Wartolowska, Michael J Thrippleton, Marco Duering, Julie Staals, Martin Middeke, Elisabeth André, Bo Norrving, Marie-Germaine Bousser, Ulrich Mansmann, Peter M Rothwell, Fergus N Doubal, Robert van Oostenbrugge, Geert Jan Biessels, Alastair JS Webb, Joanna M Wardlaw and Martin Dichgans in European Stroke Journal
