# Supplementary material for: The EffecTs of Amlodipine and other Blood PREssure Lowering Agents on Microvascular FuncTion in Small Vessel Diseases (TREAT-SVDs) trial: Study protocol for a randomised crossover trial
Source: Eur Stroke J. 2022 Dec 16;8(1):387–97. doi: 10.1177/23969873221143570 (PMC10069218; doi:10.1177/23969873221143570)
Supplement: sj-docx-1-eso-10.1177_23969873221143570 – Supplemental material for The EffecTs of Amlodipine and other Blood PREssure Lowering Agents on Microvascular FuncTion in Small Vessel Diseases (TREAT-SVDs) trial: Study protocol for a randomised crossover trial [file sj-docx-1-eso-10.1177_23969873221143570.docx]

**The EffecTs of Amlodipine and other Blood PREssure Lowering Agents on Microvascular FuncTion in Small Vessel Diseases (TREAT-SVDs) trial:**

**Study protocol for a randomised crossover trial**

Anna Kopczak^1^, Michael Stringer^2^, Hilde van den Brink^3^, Danielle Kerkhofs^4^, Gordon Blair^2^, Maud van Dinther^4^, Laurien Onkenhout^3^, Karolina Wartolowska^5^, Michael J. Thrippleton^2^, Marco Duering^1,6^, Julie Staals^4^, Martin Middeke^7^, Elisabeth André^8^, Bo Norrving^9^,

Marie-Germaine Bousser^10^, Ulrich Mansmann^11^, Peter M Rothwell^5^, Fergus Doubal^2^,

Robert van Oostenbrugge^4^, Geert Jan Biessels^3^, Alastair JS Webb^5^, Joanna Wardlaw^2,12^,

Martin Dichgans^1,13,14^

on behalf of the SVDs@target consortium

**Supplemental Material**

**Supplemental Methods**

**Choice of comparators**

The rationale for the choice of specific antihypertensive agents in TREAT-SVDs is substantiated by previous findings from clinical trials on amlodipine, losartan, and atenolol. In the blood pressure lowering arm of the Anglo-Scandinavian Cardiac Outcomes Trial (ASCOT-BPLA), patients treated with an amlodipine-based regimen suffered less often from stroke than patients treated with an atenolol-based regimen.^1^ Another study, the Losartan Intervention For Endpoint reduction in hypertension (LIFE) study, compared losartan with atenolol in hypertensive patients and showed a lower incidence of stroke in the losartan group when compared with the atenolol group.^2^ However, in both trials only 8-11% of study participants had a stroke prior to study inclusion. Hence, it is unclear if the results can be transferred to patients with stroke as a whole and more specifically to patients with SVDs. In addition, there is no information on blood pressure variability (BPv) available in both trials.

A meta-analysis on the effect of antihypertensive drug classes on BPv and stroke risk showed that variation in systolic BP was reduced by calcium channel blockers, and increased by angiotensin-receptor blockers and beta-blockers in ascending order.^3^ Against this background, we hypothesised that the calcium channel blocker amlodipine would have a beneficial effect on cerebrovascular reactivity and BPv when compared to either the angiotensin-receptor blocker losartan or the beta-blocker atenolol. Given the findings in the LIFE study, we further hypothesised that losartan would have a beneficial effect when compared to atenolol.”

**Interventions**

Patients stop taking their regular antihypertensive medication for a two-week run-in phase. Patients with coronary artery disease previously treated with beta-blockers are instructed to lower the dosage of the beta-blocker to half of the original dose for the first week of the run-in phase and to stop beta-blocker intake at the beginning of the second week of the run-in phase.

This tapering regimen is independent from the previous dosage of the beta-blocker and from the specific beta-blocker prescribed.

At the end of the run-in phase, the primary and secondary outcome measures are assessed while not taking any antihypertensive drugs. These measures serve as reference for the subsequent examination of drug effects. On the following day, patients start taking the first of the three trial drugs according to the randomised sequence of treatment allocation. Antihypertensive treatment commences with a standard dose of amlodipine, losartan, or atenolol.

Switching between study drugs is done directly without washout. This approach prevents a potential rebound phenomenon with discontinuation of atenolol and does not interfere with the primary and secondary outcome measures as these measures are assessed in the last week of monotherapy thus avoiding carry-over effects. Rescue medication does not automatically continue on the subsequent drug.

Trial medication may be increased up to 10mg amlodipine, 100mg losartan or 100mg atenolol to reach the target blood pressure (BP). Systolic BP (SBP) values of ≥140mmHg and ≤160mmHg as well as diastolic BP (DBP) values ≥90mmHg and ≤110mmHg will be tolerated during the treatment phase. Patients who show persistently elevated BP (SBP >160mmHg or DBP >110mmHg) measured on at least three days within one week will receive rescue medication. In case of low BP or orthostatic symptoms, trial drugs can be reduced to half of the standard dosage (i.e., 2.5mg amlodipine, 25mg losartan, and 25mg atenolol).

Upon trial completion, patients are put back on their original BP lowering medication while also informing participants and their general practitioner of which medication achieved the greatest BP reduction. Patients with coronary artery disease, who receive atenolol as the last trial drug, are instructed to gradually reduce atenolol according to the tapering regimen detailed above. If study physicians and patients decide to continue with one of the three antihypertensive drugs administered during the trial, the respective antihypertensive medication is prescribed after consulting the general practitioner.

**Rescue medication**

In TREAT-SVDs, thiazide or thiazide-like diuretics serve as rescue medication. The specific choice of drug (e.g. hydrochlorothiazide or bendroflumethiazide) depends on the clinical routine at individual study sites. Rescue medication is administered in standard dosage. It can be applied during the whole study period including the run-in phase.

**Previous and concomitant medication**

Any medication with known contraindication to the antihypertensive trial medication according to the respective product information is not allowed during the trial period of 14 weeks. Study participants are prohibited to take concomitant antihypertensive medication other than the trial drug and rescue medication. Medications known to potentially interact with trial drugs such as potassium-sparing agents, potassium supplements, non-steroidal anti-inflammatory drugs and strong or moderate CYP3A4 inhibitors or inducers shall only be used if deemed mandatory and with the lowest possible dosage. Intake of any concomitant medication is documented. As statins might interfere with cerebrovascular reactivity (CVR),^4^ statin dosage and the statin itself should not be changed during the trial. Changes in statin intake are assessed and documented during the trial period.

**BP measurements**

During the whole trial period of 14 weeks BP is monitored telemetrically with a dedicated BP device (Tel-O-Graph® GSM plus; I.E.M. GmbH, Germany). The device has been certified according to the European standard ISO 81060–2:2009 and is graded A/A by the British Hypertension Society. Data are transferred to a central database via mobile phone networks. Patients are instructed by a manual how to perform the BP measurement correctly. The manual is distributed together with the informed consent form.

**Laboratory investigations**

Laboratory investigations are performed at every visit. They include i) clinical chemistry laboratory values, i.e., sodium, potassium, glucose, urea nitrogen, creatinine, calcium, albumin, aspartate aminotransferase (AST), alanine aminotransferase (ALT), gamma-glutamyl transferase (GGT), alkaline phosphatase (AP), and ii) haematological laboratory values, i.e., haemoglobin, white blood cell count and platelet count. Before the run-in phase starts, HbA1c or fasting glucose, total cholesterol, HDL-cholesterol, LDL-cholesterol, and triglycerides are measured to assess the cardiovascular risk profile. A pregnancy test is performed in women of childbearing potential.

**FACS analysis (optional)**

To determine the profile of immune cells fresh venous blood samples are collected at baseline in cell preparation tubes (2 x 8ml). Isolation of immune cell subpopulations is done by flow cytometry after standard preparation of the samples for Fluorescence-activated cell sorting (FACS) analysis. Patients with systemic inflammatory disease (such as irritable bowel syndrome, inflammatory rheumatic diseases, and vasculitis), patients who use immunomodulatory/immunosuppressive drugs and patients with signs of intercurrent infections, temperature >38.0°C or use of antibiotics during the last seven days before blood sampling are excluded from FACS analysis.

For later FACS analysis, c-reactive protein (CRP) is measured at baseline. Peripheral blood mononuclear cells (PBMC’s) are collected the same day by density centrifugation of blood samples. The PBMC fraction is stored at -80°C at each study site. All frozen material is sent to the University of Maastricht for centrally performed flow cytometry.

**Harmonisation of assessments in the SVDs@target consortium**

TREAT-SVDs is part of an EU-funded project (SVDs@target) that further includes two observational studies: Zoom@SVDs^528^ and INVESTIGATE-SVDs.^6^ Efforts are made to harmonise study procedures including clinical assessments, magnetic resonance imaging (MRI) protocols, CVR and BP measurements, blood sampling for FACS analysis, data management, and data analysis.

**Co-enrolment**

Co-enrolment in an observational research study is possible as long as this would not confound TREAT-SVDs results. Participation in another randomised controlled trial is not possible.

**Data collection methods**

Research staff collect all baseline data on demographics, comorbidities, medical history, and treatments from participants and their medical records. Outcome measures are assessed with the pseudonymised patient ID. There are no provisions for ancillary and post-trial care given that the intervention tested is available in standard clinical practice.

**Data management**

The trial software has a user and role concept that can be adjusted on a trial-specific basis. The database is integrated into a general IT infrastructure and safety concept with a firewall and backup system for daily backup. After completion and cleaning of data, the database will be locked and the data exported for statistical analysis.

All written records are kept in a secure storage area with limited access. Clinical information will not be released without written permission of the participant, and research staff may not disclose or use for any purpose other than performance of the study, any data, record, or other unpublished, confidential information disclosed to them for the purpose of the study. All research staff must comply with the requirements of the Data Protection Act 1998 (applicable before 2018) and with the requirements of the General Data Protection Regulation (GDPR) for the EU and the Data Protection Act 2018 for the UK (both applicable since 2018) with regard to the collection, storage, processing and disclosure of personal information and uphold the Act’s core principles. Data completeness, range, and consistency checks are performed regularly. Incomplete data are chased by the data managers and the sponsor. Documentation about TREAT-SVDs data management procedures can be obtained from the corresponding author upon reasonable request.

**Statistical analysis**

The linear mixed effect (LME) model will be used to perform sensitivity analyses adjusting for age, sex, BP, BP variability, and other relevant baseline characteristics.

Specifically, we will compare change in CVR in NAWM after amlodipine treatment, change in CVR in NAWM after losartan treatment, and change in CVR in NAWM after atenolol treatment as primary endpoint. As statistical method, the closed testing procedure will be used that adjusts for multiple comparisons: If a general test on different effects between the three treatments is significant on level α, all paired treatment comparisons can be performed on level α without inflating the overall error rate. This means that the first step will be to analyse whether there is any significant difference between the three drugs on change in CVR in NAWM. This global test will be performed by a likelihood ratio test comparing two LME models, one with and one without treatment information. In case of violation of the assumption for the crossover, a simple parallel group comparison for the first periods of the three sequences will be performed.

If this global test shows significant differences between the trial drugs, the different pairs of drugs will be tested (amlodipine vs. losartan, amlodipine vs. atenolol, losartan vs. atenolol). If either the comparison amlodipine vs. losartan or the comparison amlodipine vs. atenolol shows a significant difference in favour of amlodipine, the primary study hypothesis will be considered positive. If the comparison losartan vs. atenolol shows a significant difference in favour of losartan, the secondary study hypothesis will be considered positive. This approach will be used for the primary endpoint and for the secondary endpoints.

In additional sensitivity analyses, we will account for the time interval between stroke and study inclusion and for the presence of MRI features of SVDs. Further sensitivity analyses will be performed to account for patients with cognitive impairment and with confluent deep white matter hyperintensities on MRI who also show signs of mixed dementia or other dementia subtypes.

**Withdrawal**

A patient can be withdrawn from the trial for the following reasons: (i) withdrawal of informed consent, (ii) need of acute medical treatment that significantly interferes with the trial intervention, (iii) occurrence of exclusion criteria during intervention such as eGFR <35ml/min, atrioventricular block III°, sick sinus syndrome, heart failure NYHA ≥III, resting heart rate <50/min, > 5-fold increased levels of transaminases (e.g. ALT >250U/l, AST >250U/l, GGT >300U/l depending on sex and local reference ranges), start to use monoamine oxidase (MAO)-A-blockers, start to use simvastatin >20mg/d, severe hypercalcaemia >3mmol/l (total calcium level), hypokalaemia <3mmol/l despite adequate replacement therapy, hyponatraemia <130mmol/l despite adequate replacement therapy, symptomatic gout with hyperuricaemia >6.5mg/dl, and pregnancy. Patients with persistent asymptomatic hypertension (SBP >180mmHg or DBP >120mmHg) on at least three days within one week despite current treatment with maximum-dose rescue medication during the run-in phase (2 weeks) are withdrawn from the trial. During the treatment phase (12 weeks), patients with persistent asymptomatic hypertension (SBP >160mmHg or DBP >110mmHg) on at least three days within one week despite current treatment with maximum-dose rescue medication and maximum-dose trial drug are withdrawn.

When a patient has a specific non-tolerable side effect of a trial drug, this patient is switched to the next trial drug according to the treatment sequence that has been assigned by randomisation. Patients are not allowed to change their assigned treatment sequence for any reason.

**Legislation and guidelines**

TREAT-SVDs is conducted in accordance with the published principles of the guidelines for Good Clinical Practice (ICH-GCP) and the relevant national legislation. These principles cover, amongst other aspects, ethics committee procedures, the obtaining of informed consent from trial subjects, adherence to the trial protocol, administrative documentation, and documentation regarding the IMP, data collection, trial subjects’ medical records (source documents), documentation and reporting of adverse events, preparation for inspections and audits, and the archiving of trial documentation. All investigators and other staff directly concerned with the trial will be informed that domestic and foreign supervisory bodies, the competent federal authorities and authorised representatives of the sponsor have the right to review trial documentation and the trial subjects’ medical records at any time.

**Classification as phase IIIb clinical trial**

The safety profile of all three trial drugs is well established and all three drugs have been approved for market for the treatment of hypertension. However, TREAT-SVDs is testing the efficacy of these drugs on CVR, which has so far not been addressed in a clinical trial. Since the trial is testing the efficacy of drugs that have been approved for market towards a new clinical endpoint, TREAT-SVDs is classified as phase IIIb trial.

**Amendments to the trial protocol**

To ensure that comparable conditions are achieved as far as possible at individual trial sites and in the interest of a consistent and valid data analysis, changes to the provisions of the trial protocol are not planned. In exceptional cases, however, changes may be made to the trial protocol. Such changes can only be made if agreed by the sponsor, sponsor’s representative, the biometrician, and the Steering Committee. Any changes to the trial procedures must be made in writing and must be documented with reasons and signed by all lead authors on the trial protocol.

Amendments made in accordance with § 10 Secs. 1 and 4 GCP Regulations that require approval are submitted to the ethics committee and the supreme federal authority. Exceptions to this are amendments made to avoid immediate dangers.

The current protocol is version 4.0, created on 03 June 2022, in Germany and so far still version 3.0, created 25 February 2019, in the Netherlands and in the UK. All protocol updates since version 2.0, created 20 September 2016, which was implemented before randomisation began, have been approved by the sponsor and responsible Research Ethics Committees and also communicated to investigators and trial registries.

**Dissemination policy**

The current protocol report complies with the SPIRIT (Standard Protocol Items: Recommendations for Interventional Trials) 2013 reporting guideline.^7,8^ On completion of the trial, the data will be analysed and tabulated, a clinical study report will be prepared in accordance with Good Clinical Practice (GCP) guidelines, and a manuscript for publication will be prepared in accordance with Consolidated Standards of Reporting Trials (CONSORT) guidelines. Active collaborators included in the delegation logs at sites that have recruited participants will be included in any listing of collaborators. The primary trial manuscript will be approved by the Clinical Steering Committee of SVDs@target before submission for publication. Results will be disseminated to the patient organisation Stroke Alliance for Europe (SAFE; https:/safestroke.eu), to study participants (if requested), via social media channels (twitter), and online (https://www.svds-at-target.eu/).

**Data sharing**

Ownership of the data arising from TREAT-SVDs resides with the Steering Committee. Access to the datasets generated or analysed during TREAT-SVDs will be available upon reasonable request compliant with general data protection regulation (GDPR) after the publication of the main results consistent with recent guidance.^9^ Access will be controlled by the chief investigator with approval of the Steering Committee.

**Roles and responsibilities**

TREAT-SVDs is sponsored by the Klinikum der Universität München, Marchioninistr. 15, D-81377 Munich, Germany.

**References**

1. Dahlöf B, Sever PS, Poulter NR, et al. Prevention of cardiovascular events with an antihypertensive regimen of amlodipine adding perindopril as required versus atenolol adding bendroflumethiazide as required, in the Anglo-Scandinavian Cardiac Outcomes Trial-Blood Pressure Lowering Arm (ASCOT-BPLA): a multicentre randomised controlled trial. *Lancet.* 2005;366:895-906.

2. Dahlöf B, Devereux RB, Kjeldsen SE, et al. Cardiovascular morbidity and mortality in the Losartan Intervention For Endpoint reduction in hypertension study (LIFE): a randomised trial against atenolol. *Lancet.* 2002;359:995-1003.

3. Webb AJ, Fischer U, Mehta Z, Rothwell PM. Effects of antihypertensive-drug class on interindividual variation in blood pressure and risk of stroke: a systematic review and meta-analysis. *Lancet.* 2010;375:906-915.

4. Giannopoulos S, Katsanos AH, Tsivgoulis G, Marshall RS. Statins and cerebral hemodynamics. *J Cereb Blood Flow Metab.* 2012;32:1973-1976.

5. van den Brink H, Kopczak A, Arts T, et al. Zooming in on cerebral small vessel function in small vessel diseases with 7T MRI: rationale and design of the “ZOOM@ SVDs” study. *Cerebral Circulation-Cognition and Behavior* 2021;2:100013.

6. Blair GW, Stringer MS, Thrippleton MJ, et al. Imaging neurovascular, endothelial and structural integrity in preparation to treat small vessel diseases. The INVESTIGATE-SVDs study protocol. Part of the SVDs@ Target project. *Cerebral Circulation-Cognition and Behavior.* 2021;2:100020.

7. Chan AW, Tetzlaff JM, Altman DG, et al. SPIRIT 2013 statement: defining standard protocol items for clinical trials. *Ann Intern Med* 2013;158:200-207.

8. Chan AW, Tetzlaff JM, Gøtzsche PC, et al. SPIRIT 2013 explanation and elaboration: guidance for protocols of clinical trials. *BMJ.* 2013;346:e7586.

9. Tudur Smith C, Hopkins C, Sydes MR, et al. How should individual participant data (IPD) from publicly funded clinical trials be shared? *BMC medicine.* 2015;13:298.

**Supplemental Acknowledgments**

The TREAT-SVDs collaborators are:

**Ludwig-Maximilians-Universität Munich, Institute for Stroke and Dementia Research, Germany**: Prof. Martin Dichgans, Dr. Anna Kopczak, Dr. Karin Waegemann, Prof. Marco Düring, Mr. Daniel Janowitz, Dr. Maria Kaffe, Mr. Mathias Hübner, Mrs. Sandra Hein, Dr. Anna Dewenter

**University of Oxford, Nuffield Department of Clinical Neurosciences,** **UK**: Prof. Peter Rothwell, Prof. Alastair Webb, Dr. Karolina Wartolowska, Mrs. Luise Silver, Mrs. Josie Brooks

**University of Edinburgh, Neuroimaging Sciences and Brain Research Imaging Centre,** **UK**: Prof. Joanna Wardlaw, Dr. Fergus Doubal, Dr. Michael Stringer, Dr. Michael Thrippleton, Dr. Gordon Blair, Dr. Carmen Reyes Arteaga, Mrs. Daniela Jamie Garcia, Mrs. Iona Hamilton, Mrs. Rosalind Brown, Mrs. Una Clancy

**University of Utrecht, Utrecht Brain Center Robert Magnus,** **The Netherlands**: Prof. Geert-Jan Biessels, Mrs. Hilde van den Brink, Dr. Laurien Onkenhout, Dr. Tine Arts, Mrs. Manja Litjens

**University of Maastricht, Department of Neurology,** **The Netherlands**: Prof. Robert van Oostenbrugge, Dr. Julie Staals, Dr. Danielle Kerkhoffs, Mrs. Maud van Dinther

**University of Glasgow, School of Psychology & Neuroscience,** **UK**: Prof. Keith Muir

**University of Leiden, Department of Clinical Genetics,** **The Netherlands**: Prof. Saskia Lesnik-Oberstein, Dr. Remco Hack

**Supplemental Figures**


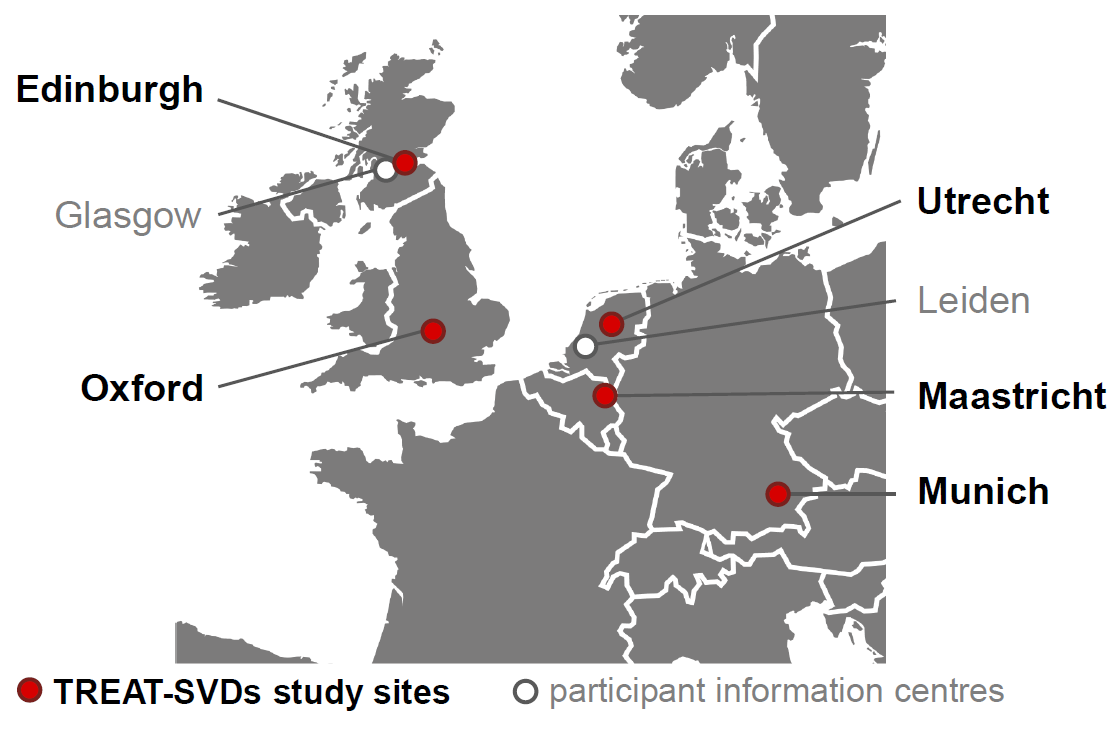


**Supplemental Figure 1: Study sites and participant information centres involved in the TREAT-SVDs trial.** TREAT-SVDs is conducted at five study sites in Europe. Glasgow (UK) and Leiden (The Netherlands) serve as participant information centres for patients with CADASIL (Cerebral Autosomal Dominant Arteriopathy with Subcortical Infarcts and Leukoencephalopathy).

**Supplemental Tables**

|  | T1w | FLAIR | T2w | SWI | dMRI | CVR |
| --- | --- | --- | --- | --- | --- | --- |
| Sequence | MPRAGE  (3D IR-sGRE) | SPACE  (3D RARE) | SPACE  (3D RARE) | 3D sGRE | 2D spin echo-EPI | 2D GRE-EPI |
| Voxel size | 1 x 1 x 1 | 1 x 1 x 1 | 0.9 x 0.9 x 0.9 | 0.6 x 0.6 x 3 |  |  |
| In-plane resolution |  |  |  |  | 2 x 2 | 2.5 x 2.5 |
| Slice thickness |  |  |  |  | 2 | 2.5 |
| Inter-slice gap |  |  |  |  | 0% | 0% |
| TR | 2500 | 5000 | 3200 | 28 | 4300 | 3000 |
| TE | 4.37 | 388 | 408 | 20 | 74 | 30 |
| TI | 1100 | 1800 | - | - | - | - |
| Flip angle | 7 | - | - | 9 | - | 90 |
| Acquisition time | 3:45 | 5:57 | 3:42 | 4:02 | 11:12 | 12:30 |
| Other | *R* = 3 | *R* = 3 | *R* = 2 x 2 | *R* = 2 | *R* = 2, MB = 2,  14xb=0s/mm^2^,  3xb=200s/mm^2^,  6xb=500s/mm^2^,  64xb=1000s/mm^2^,  64xb=2000s/mm^2^,  (3xb=0s/mm^2^ with reversed phase encoding) | *R* = 2 |

**Supplemental Table 1. Acquisition parameters for the TREAT-SVDs imaging protocol of visit 1**

This imaging protocol of visit 1 results in a total scanning time of 00:42:24. The first MRI scan is done at the end of the wash-out phase. Voxel size for 3D sequences, and in-plane resolution and slice thickness for 2D sequences in mm. TR/TE/TI all in milliseconds. Acquisition time in minutes and seconds. Flip angle in degrees. CVR = cerebrovascular reactivity; dMRI = Diffusion imaging; EPI = echo-planar imaging; FLAIR = Fluid-attenuated inversion recovery; GRE-EPI = Gradient-echo echo-planar imaging; IR = Inversion recovery; MB = multiband acceleration factor; MPRAGE = Magnetisation-prepared rapid acquisition with gradient echo; R = parallel imaging acceleration factor; sGRE = Spoiled gradient recalled echo; SPACE = Sampling perfection with application-optimised contrast using different flip-angle evolution; SWI = Susceptibility weighted imaging; TE = Echo time; TI = Inversion time; TR = Repetition time.

|  | T1w | FLAIR | FLASH | dMRI | CVR |
| --- | --- | --- | --- | --- | --- |
| Sequence | MPRAGE  (3D IR-sGRE) | SPACE  (3D RARE) | sGRE | 2D spin echo-EPI | 2D GRE-EPI |
| Voxel size | 1 x 1 x 1 | 1 x 1 x 1 | 1 x 1 x 5 |  |  |
| In-plane resolution |  |  |  | 2 x 2 | 2.5 x 2.5 |
| Slice thickness |  |  |  | 2 | 2.5 |
| Inter-slice gap |  |  |  | 0% | 0% |
| TR | 2500 | 5000 | 742 | 4300 | 3000 |
| TE | 4.37 | 388 | 19.9 | 74 | 30 |
| TI | 1100 | 1800 | - | - | - |
| Flip angle | 7 | - | 20 | - | 90 |
| Acquisition time | 3:45 | 5:57 | 1:32 | 5:32 | 12:30 |
| Other | *R* = 3 | *R* = 3 | *R* = 2 | *R* = 2, MB = 2,  8xb=0s/mm^2^,  64xb=1000s/mm^2^,  (3xb=0s/mm^2^ with reversed phase encoding) | *R* = 2 |

**Supplemental Table 2. Acquisition parameters for the TREAT-SVDs imaging protocol of visits 2 - 4**

This imaging protocol of visits 2 - 4 results in a total scanning time of 00:30:32. MRI scans are performed at the end of the four-week period of monotherapy with one of the three trial drugs.

Voxel size for 3D sequences, and in-plane resolution and slice thickness for 2D sequences in mm. TR/TE/TI all in milliseconds. Acquisition time in minutes and seconds. Flip angle in degrees. CVR = cerebrovascular reactivity; dMRI = Diffusion imaging; EPI = echo-planar imaging; FLAIR = Fluid-attenuated inversion recovery; FLASH = Fast Low Angle Shot; GRE-EPI = Gradient-echo echo-planar imaging; IR = Inversion recovery; MB = multiband acceleration factor; MPRAGE = Magnetisation-prepared rapid acquisition with gradient echo; R = parallel imaging acceleration factor; sGRE = Spoiled gradient recalled echo; SPACE = Sampling perfection with application-optimised contrast using different flip-angle evolution; TE = Echo time; TI = Inversion time; TR = Repetition time.
